# Researchers’ roadblocks to including people with intellectual and developmental disabilities (DD) in research: Translational science and I/DD program leaders insights

**DOI:** 10.1017/cts.2025.10213

**Published:** 2025-12-04

**Authors:** Karen Bonuck, Patrick George, Mark Harniss, Frank Meeuwis, Suzannah Iadarola

**Affiliations:** 1 Professor, Department of Family and Social Medicine, Co-Director University Center of Excellence in Developmental Disabilities, Albert Einstein College of Medicine-Montefiore Medical Center, Bronx, NY, USA; 2 Study Coordinator, Department of Family and Social Medicine, Albert Einstein College of Medicine-Montefiore Medical Center, Bronx, NY, USA; 3 Professor, Department of Rehabilitation Medicine, Director of the Center for Technology and Disability Studies; Director of the UW Disability Studies Program, University of Washington, Seattle, WA, USA; 4 Consultant, Albert Einstein College of Medicine-Montefiore Medical Center, Bronx, NY, USA; 5 Associate Professor, Department of Pediatrics and Public Health, Strong Center for Developmental Disabilities, University of Rochester, Rochester, NY, USA

**Keywords:** Disability, developmental disability, underrepresented, inclusion, qualitative

## Abstract

People with disabilities in the US are now a health disparities population. Though 25% of US adults have a disability, only 5% of medical research grants are disability related. Knowledge about researchers’ perceived barriers to including people with disabilities in research has focused on a single disability/condition and thus has limited translational science applications. Our CTSA’s Disability as Difference: Reducing Researcher Roadblocks (D2/R3) project examined such roadblocks towards inclusion of people with intellectual and developmental disabilities (I/DD). I/DDs are broad, heterogeneous conditions that originate in childhood, have varying impact and function, and persist throughout the lifespan. Strategies that mitigate their under-representation in research will likely have general applicability to all disabilities. In D2/R3’s first phase we conducted semi-structured interviews with translational science and I/DD program leaders at ten US institutions about perceived barriers and facilitators to including people with I/DD in research. Interviews were held with 25 individuals from partnering Intellectual and Developmental Disabilities Research Centers, University Centers for Excellence in Developmental Disabilities, and Clinical and Translational Science Award programs. Collaborative thematic coding identified key themes as: attitudinal barriers (e.g., assumptions about consent capacity), logistical barriers (e.g., accommodation costs), health disparities, and generalizability concerns. Findings informed development of a survey based on Prosci’s ADKAR® model of change management’s five components: Awareness, Desire, Knowledge, Ability and Reinforcement. Exclusion appears to stem from researchers’ lack of awareness, misconceptions, and knowledge gaps rather than insurmountable obstacles.

## Introduction

People with disabilities were designated a health disparities population in 2023. In making the designation, the NIH cited myriad barriers, unmet needs, and lack of effective interventions and expected that it would “help to improve access to healthcare and health outcomes for all people [1].” Indeed, evidence cited for the designation aligns with translational science principles, such as prioritizing research addressing unmet patient and population health needs [2] and diversifying clinical trial recruitment [3]. The NIH awarded 248,454 research project grants from 2018 to 2022 [4]; just 4% (9481) were disability-related R01 equivalents [5]. With ≈25% of US adults identifying as having a disability [6], this underrepresentation yields inadequate evidence on which to inform clinical management, leading to suboptimal care and exacerbates disparities [7].

While the designation is a step forward, realizing its goals requires explicitly addressing historical barriers to underrepresentation. Researchers exclude this population from studies due to assumptions about safety risks/heightened vulnerability [7], difficulties accessing the population [8,9], and consent capacity [10,11]. Structural barriers include inaccessible protocols (e.g., not in plain language) and physical spaces, and limited funding to develop new methods and testing protocols [7,12,13]. For people with disabilities, this has resulted in mistrust of research due to negative healthcare experiences [8,9,13,14]. Researchers also often lack the knowledge and skills to engage people with DD [9,15]. These factors have led to broad, poorly justified exclusion criteria, resulting in people with disabilities – and especially those with DDs – marked absence from scientific research [10,11].

Little is known about how researcher knowledge and perceptions contribute to barriers for people with disabilities and people with DD, compared to recruitment of other underrepresented groups. A 2023 scoping review of recruiting people with disabilities into clinical trials found that only 2 of 56 papers addressed general versus specific disabilities [7]. Given that people with disabilities “often experience a wide and varying range of health conditions [1],” this reductionist, condition-specific approach to disability inclusion has limited utility for bringing about the transformational change sought by translational science [16].

To address this limitation, our CTSA initiated the *Disability as Difference: Reducing Researcher Roadblocks* (D2/R3) study. D2/R3 surveyed researchers about their knowledge, attitudes, biases, and perceptions regarding the inclusion of people with DD in research. Findings were used to develop and trial an intervention designed to reduce these roadblocks. Einstein-Montefiore’s CTSA and partners lead D2/R3, which includes 10 US institutions with a CTSA, Intellectual and Developmental Disabilities Research Center for basic/clinical research, and University Center for Excellence in Developmental Disabilities Education, Research, and Service programs. Its focus is researchers – investigators, research assistants, clinical research coordinators, etc. – who study conditions that disproportionately affect adults with disabilities and with DD, for example, diabetes and heart disease – but do not specifically conduct research about adults with disabilities. As detailed elsewhere, D2/R3 began by exploring roadblocks to researchers’ inclusion of adults with DD, rather than all disabilities. DDs are characterized by childhood onset, lifelong impact, and often non-visible features, distinguishing them from disabilities typically acquired with age (i.e., wheelchair as the universal icon for disability). Beginning with this more holistic conceptualization of disability increases the likelihood that D2/R3 findings can generalize to all disabilities, but not vice versa [16].

This paper reports on our interviews with leadership from the 10 sites’ CTSA, Intellectual and Developmental Disabilities Research, and University Center of Excellence in Developmental Disabilities programs that a) elicited their views on how researchers perceive the barriers and facilitators to People with Developmental Disabilities (PWDDs) participation in research and b) informed development of the D2/R3 researcher survey noted above. The study’s conceptual framework for reducing researcher roadblocks is Grounded in Prosci’s ADKAR® model of change management [17], which comprises five elements: **A**wareness of the need for change, **D**esire to enact or support change, **K**nowledge of how to change, **A**bility to implement desired skills and behaviors, and **R**einforcement to sustain change. A prime example of ADKAR® in practice is hotels advising guests about the ecological benefits of reusing their towels (i.e., awareness/desire) and providing them with a hook for reusing towels (knowledge/ability/reinforcement).

Terminology: DDs refer to broad disabilities that can be intellectual, physical, or both. Intellectual disability is the term used when both an intellectual disability and another disability are present. All DDs begin in childhood (<22 years), impact physical, cognitive, and/or social function and are often lifelong. This project focuses on the broader category of DDs.

## Methods

### Setting/participants

The sites included Einstein College of Medicine/Montefiore (New York City), Kennedy Kreiger Institute (Baltimore, MD), University of California (Davis), University of California (Los Angeles), University of Iowa, University of North Carolina, University of Rochester Medical Center, University of Washington, and Vanderbilt University. Interviews were then arranged with each site’s program leaders to build rapport and elicit their views on people with DD underrepresentation in research to inform survey development (see above). One site declined to have their data used in this reporting.

### Interviews

Program leaders from each site participated in online, semi-structured interviews lasting ≈60 minutes, conducted by KB and PG. Reminder emails were sent a day before the scheduled interview. These interviews were introductory and exploratory in nature, conducted for the purpose of survey development rather than research. As such, formal informed consent for research participation was not obtained at the time. Audio recordings were of insufficient quality for verbatim transcription and analysis. Key insights were captured through detailed notes and paraphrasing of verbal exchanges.

### Analysis

The primary analysis comprised two interconnected phases: a) collaborative coding of qualitative interview excerpts by the authorship team and b) application of insights from excerpts and themes to craft D2/R3 survey items aligned with the ADKAR® model.

### Collaborative coding

All authors developed an initial concept map of questions that excerpts encompassed: (1) What are the barriers? (*identification*), (2) What are their consequences? (*implications*), and (3) How could they be eliminated? (*solutions*). With this framing, we applied thematic analysis to the paraphrased excerpts. Authors reached a consensus on codes and their definitions (below) before independently coding the excerpts. Authors then reconvened across several sessions to reconcile differences, refine code definitions reflexively, and recode the paraphrased excerpts independently. As noted in a leading qualitative data analysis sourcebook: “For all approaches to coding, several codes will change and develop as field experience continues.” (p. 86, Miles, Huberman and Saldana, 2014) [18].

Authors’ Positionality Statement – As a group, our roles encompass: Self-Advocate (adult with DD), UCEDD program leadership, DD researchers, and family members of persons with DD. As with any research, especially qualitative, positionality and perceptions may have influenced the coding. Leadership of partnering translational science and DD programs were approached for interviews.

### Thematic code definitions

Authors reached a consensus on the following codes:

- Disparity: People with DD are underrepresentated in research; how this exacerbates health outcomes (i.e., treatments ineffective or not studied in people with DD); poor understanding of people with DD health issues, lack of access to care, etc.
- Attitudinal barriers: Assumptions of researchers, institutional review boards, and regulatory agencies regarding the capacity of people with DD to understand the study purpose, what consent entails, and to participate in research meaningfully.
- Logistical barriers: Practices of researchers that may create roadblocks from initial outreach to recruitment -> consent ->participation->retention.
- Generalizability: Validity of research is reduced when target populations with the condition of interest are excluded from studies.
- Solutions: Strategies used/suggested by researchers to reduce barriers and make research more equitable, for example, progressive quizzing and consent process adaptations.

### *Excerpts’ application to ADKAR*® *model and items*

Our applying of the ADKAR theoretical framework used in D2/R3’s initial (Aim 1) survey to this qualitative analysis emerged after analysis of the paraphrased excerpts. These survey items were informed by the literature, dialog with adults with developmental disabilities and *ADKAR*®. Thus, we identified if and how excerpts: a) related to ADKAR elements and b) directly informed the D2/R3 study’s creation of specific survey items (in conjunction with the literature). Two members of the core D2/R3 team (KB and PG) mapped paraphrased excerpts to corresponding ADKAR concepts and Year 1 survey questions (see Table 1). Note, as this phase was not conducted under a research protocol, it was completed by study personnel (inclusive of KB, PG, and FM).

**Table 1.** Coding analysis

Excerpts, by thematic code (total # in code)	Excerpt, by ADKAR® element	Excerpt, as applied to D2/R3 survey (relevant questions #)
Attitudinal barriers (4/8 excerpts shown)		
Historical explicit and implicit exclusions (e.g., consent language) --> assumption that people with DD are “not valuable sources of data”	Awareness	Knowledge of how to adapt consenting (Q4) Ability to adapt (Q5)
Explore why researchers think it is difficult to engage people with DD in research	Awareness	Miscellaneous
Researchers assume that people with DD are hard to consent	Awareness Desire Reinforcement	Knowledge of how to adapt consenting (Q4) Ability to adapt (Q5)
Perceive people with DD barriers to being a participant: medical accommodations, monitoring adverse events	Knowledge Ability	Knowledge of potential risk for people with versus without DD (Q8) Ability to any potential extra risk (Q9) Knowledge (vs. perception) of evidence likely to exclude people with DD (Q10)
Assumptions lead to researchers less likely to engage with people with DD	Reinforcement	Reinforcement – by IRBs, NIH, and journals (Q12a,b,c) Disability as Diversity – impact of NIH designation (13a,b,c)
Disparity (2/4 excerpts shown)		
Access gap for adults w IDD: medications, primary care, and mental health	Awareness Knowledge	Option: visual of disparities in NIH funding for children and adults with and without I/DD (Q1)
Recognize barriers to engaging PWDD and health gap	Awareness Knowledge	Awareness of health and research disparities (optional disparity visual +Q1) Knowledge/recognition of barriers re: consent (Q4), design (Q6), risk (Q8), and eligibility (Q10)
People most affected by health disparities are locked out of health research*	Awareness	Option: visual of disparities in NIH funding for children and adults with and without DD (Q1)
Gap in DD services for adult: meds, mental health, primary care, etc.	Awareness	NA
Logistical barriers (5/14 excerpts shown)		
Increased accommodations need > funding (perceived barrier)	Reinforcement	Knowledge and Ability to adapt consent (Q4, Q5) and design (Q6, Q7), assuming funders approving well-justified requests for adaptations
Barriers to including people with DD in research are known--> relate to D2/R3 survey items on knowledge and perception	Awareness Knowledge	Awareness of health and research disparities (optional disparity visual +Q1)
Inclusive research > expensive, requires > time/energy from researcher perspective	Ability Reinforcement	Ability to implement accommodations (Q5, Q7) Reinforcement – by IRBs, NIH, and journals (Q12a,b,c)
“How would we accommodate… in a chair or [sic] confined to a bed?”	Ability Knowledge	Knowledge of accommodations for functional impacts (Q4, Q6) Ability to implement accommodations (Q5, Q7)
Ensure awareness of potential as research participant	Awareness	Disability as Diversity: Impact of NIH designation on “considering people with DD as suitable research participants” (Q13b)
Solutions (5/12 excerpts shown)		
Multiple recruitment pathways, for example, accessible websites, via MyChart, chronic disease registries, easier sign-ups	Knowledge Ability	Disability as Diversity: Impact of NIH designation on “recruiting people with DD for your research” (Q13c)
Simpler research language will have universal benefits	Ability Knowledge	Knowledge and Ability to adapt consent (Q4, Q5) and design (Q6, Q7)
Focus on conceptualization of research measure; construct them to be more inclusive	Ability Knowledge	Knowledge and Ability to adapt consent (Q4, Q5) and design (Q6, Q7)
Transform study spaces to be more inclusive; remote interviews	Ability	Knowledge and Ability to adapt design (Q6, Q7)
Set-aside monies for accessibility/accommodations in all grant-funded research	Ability Reinforcement	Reinforcement – by IRBs, NIH, and journals (Q12a,b,c)

After D2/R3 researchers reviewed the paraphrased excerpts and realized the richness of the data, a protocol was submitted to and approved by the Office of Human Subjects Protection at Einstein-Montefiore. Interviewees’ informed consent was obtained retroactively.

## Results

We held 10 interviews from June to August 2023 with 25 individuals from the sites’ IDDRC (*n* = 12), UCEDD (*n* = 8), and/or CTSA (*n* = 8) programs (several interviewees held dual roles).

Selected excerpts from each code are shown in Table 1, alongside their corresponding ADKAR element(s) (if applicable) and application to survey item development. Logistical barriers (*n* = 14) were the barrier most frequently assigned code, followed by solutions (*n* = 12), attitudinal barriers (*n* = 8), disparity (*n* = 4), and generalizability (*n* = 2).

Researchers’ a*ttitudinal* barriers cited by interviewees affirm findings from the literature, for example, that PWDDs are difficult to consent, “not valuable sources of data,” and that medical accommodations and adverse event monitoring are barriers. These attitudinal barriers reflect a self-reinforcing cycle of exclusion, partially driven by researcher misconceptions and inexperience about people with DD, rather than their actual limitations. For example, while several interviewees stated that researchers assume PWDDs are an “inaccessible” population, as a group, they proposed myriad solutions for recruiting them. *Logistical* barriers cited focused on accommodations, how to pay for them, and how to ensure that both people with DD and researchers know they can and should participate in research.

Not surprisingly, program leaders were highly attuned to the experience of health *disparities* and being excluded from research and its effects on the *generalizability* of findings. They offered several *solutions* to researcher roadblocks, including multiple recruitment pathways, plain language, and more inclusive research settings and measurement tools. One interviewee affirmed the value of exploring why researchers think it is hard to engage people with DD, while another cited the need for set-aside grant funding to support accessibility and accommodations.

Excerpts were well aligned with ADKAR’s five elements (second column), confirming the selection of ADKAR as the conceptual model for D2/R3. Interviewee comments also directly informed the development of the D2/R3 Perceptions survey, which was drafted with input from the larger D2/R3 project team (see acknowledgments). The D2/R3 survey, annotated by the ADKAR element, is attached as an Appendix.

## Discussion

The current state of disability research rarely centers the ideas, preferences, and inclusion of people with DD, and structural ableism in research has been named a barrier to the “right to science [18].” This study’s first aim was to elicit CTSA and/DD program leaders’ views on how researchers perceive barriers and facilitators to including PWDDs in their research. Results aligned with prior work across five themes: attitudinal and logistical barriers, disparities, generalizability, and solutions. Interviewees affirmed that: people with DD experience significant health disparities, accommodations require additional funding, and that disability crosses diagnoses and conditions. Interviews underscored researchers’ lack of awareness that people with disabilities and those with DD in particular can participate in research and are valuable sources of information. Although some members of the DD community may understand these barriers (see: *American Journal of Intellectual and Developmental Disabilities,* September 2023 issue [19]), researchers unfamiliar with disability or DD may not. Beyond awareness of whether people with DD *can* participate in research, researchers showed limited insight into *how* to best support their inclusion. Although strategies exist for meaningful individual involvement [20], structural barriers can be more difficult to address.

Additional insights from leadership interviews included the value of including people with DD in health research (e.g., generalizable study findings). Blanket assumptions about people with DDs ability to engage in research have left a dearth of knowledge concerning the health of people with disabilities. Awareness can serve as a road to understanding the value of inclusion, but systemic challenges such as IRB regulations, adverse effect monitoring, and managing consent capacities create barriers to participation for people with DD.

Our second aim was to apply interview findings to drafting the D2/R3 survey; Table 1 depicts how they were applied. Based on these findings, we included an explanatory textbox at the beginning of the survey and options for additional content before each ADKAR (i.e., awareness, desire, knowledge, ability, and reinforcement) section. We conclude that lack of knowledge likely contributes to overestimating risks and underestimating the potential benefits of including people with DD in studies. This reflects a research environment that systemically excludes people with DD, not due to insurmountable barriers but because of biases, knowledge gaps, and lack of incentives.

### Limitations

This study’s findings represent the limited perspective of IDDRC, UCEDD, and CTSA leaders at participating institutions. The participation of people with DD from those institutions in other D2/R3 activities (e.g., co-developing and reviewing the researcher survey) is not reflected here. In a subsequent phase of D2/R3, people with DD and researchers will engage in dialog to process survey findings and inform the messaging and content for a randomized controlled trial of an eLearning intervention. This collaborative approach aims to bridge the gap between researcher perceptions and the lived experiences of people with disabilities and especially those with DD, potentially leading to more inclusive and effective research practices.

## Supporting information

10.1017/cts.2025.10213.sm001 Bonuck et al. supplementary material Bonuck et al. supplementary material

## Supplementary material

The supplementary material for this article can be found at https://doi.org/10.1017/cts.2025.10213.
