# Supplementary material for: Researchers’ roadblocks to including people with intellectual and developmental disabilities (DD) in research: Translational science and I/DD program leaders insights
Source: J Clin Transl Sci. 2025 Dec 4;10(1):e2. doi: 10.1017/cts.2025.10213 (PMC12797176; doi:10.1017/cts.2025.10213)
Supplement: Bonuck et al. supplementary material [file S2059866125102136sup001.docx]

**Study Survey**

**Disability as Diversity: Reducing Researcher Roadblocks (D2/R3)**

Perceptions. Questions 1-13

**Awareness (A)**

**Introduction to Questions 1: Disparities**

Health disparities are preventable differences in the burden of disease.

**Option for click here: Question 1: Disparities**


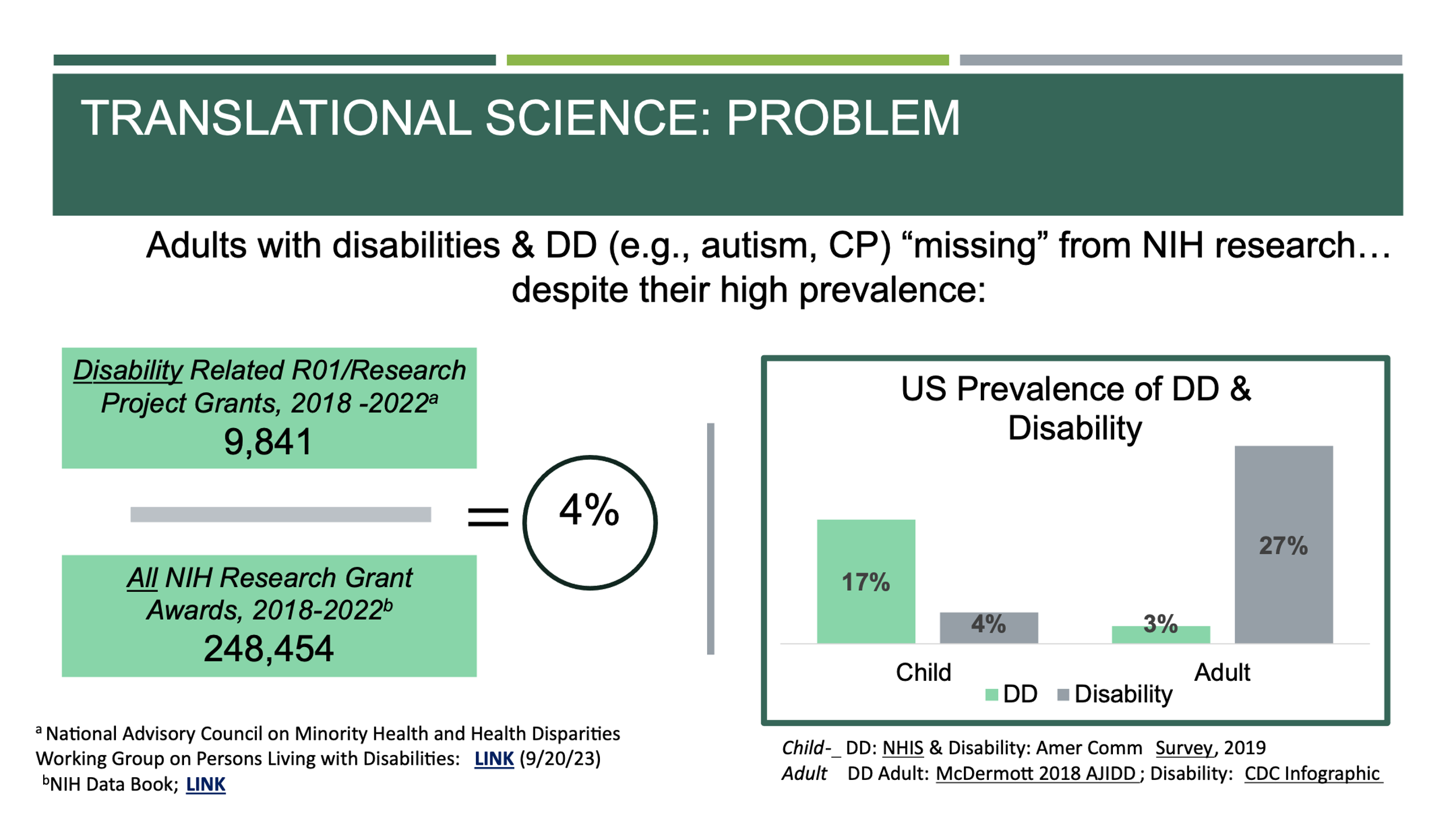


NIH Grants:

Persons with disabilities are under-represented in most biomedical and in the portfolio of NIH research. Between 2018 and 2022, **just 4%** of all NIH research grants were disability related R01s—just 9,841^a^ out of 248,454^b^.

a) National Advisory Council on Minority Health and Health Disparities Working Group on Persons Living with Disabilities: [LINK](https://nimhd.nih.gov/about/directors-corner/messages/health-disparities-population-designation.html)

b) NIH Data Book; [LINK](https://report.nih.gov/nihdatabook/category/6)

| **Question 1**   1. How aware are/**were you (if they clicked)** of the health and research disparities experienced by adults with disabilities (PWDs) and with developmental disabilities (PWDDs)?   **o** Not at all aware **o** Slightly aware **o** Somewhat aware **o** Moderately aware **o** Extremely aware |
| --- |

**DESIRE (D)**

| **Introduction to Questions 2 & 3: Inclusive Practices**  Inclusive practices often require extra time and resources, e.g., personnel, space, training, scheduling flexibility.  **Option for click here: Questions 2 & 3: Inclusive Practices**  Below are some examples of additional time or resources needed for inclusive practices:   - Communication- plain language; accessible formats, e.g., assistive technology, large dark print on white background; use internet sites compatible w screen readers; text-to-speech - Logistics- flexible timing & scheduling to address fatigue and transportation issues; extra space for mobility aid, caregiver. - Procedures- extra time & skills for blood draws - Materials-identify accommodation needs beforehand (e.g., accessible exam table), ensure they are available & research staff are trained to use them | |  |
| --- | --- | --- |
| **Questions 2 & 3: Inclusive Practices**   1. Think about the conceptualization and design phase of your research (e.g., grant development). Now, rate your **desire to take into account** how and to what extent the condition(s) you study affect PWDs/PWDDs when you conceptualize and design research:   **o** Very low **o** Low. **o** Moderate **o** High **o** Very high. **O** NA for my role   1. Now rate your **motivation to make changes needed** to engage PWD/IDD into your research?   **o** Very low **o** Low. **o** Moderate **o** High **o** Very high. **O** NA for my role  **KNOWLEDGE (K) & ABILITY (A)**  **Introduction to Questions 4 thru 11:**  Inclusive research practices can be time-consuming and resource-demanding.(Shariq, Cardoso Pinto et al. 2023) For remaining questions assume that funding agencies would approve well-justified requests for flexibility, time, and resources. Thus, answer questions with a focus on *importance*—rather than feasibility. | |  |
| **Option for click here: Questions 4 & 5: Consent**  Potential strategies for making the consent process more accessible include:  plain language, progressive quizzing for understanding, send ahead of time, alternative formats. . larger print on study instruments, verbal consent forms, utilizing plain language, closed captioning, accessible technology. Consider universal design at the outset.  **Questions 4 & 5: Consent**   1. Rate your **knowledge of how to adapt consenting,** PWD/DD whose difficulties include: cognitive, communication, visual, hearing, mobility, reading   **o** Very low **o** Low. **o** Moderate **o** High **o** Very high. **O** NA for my role   1. Rate your **ability to adapt** consenting PWD/DD with the above   **o** Very low **o** Low. **o** Moderate **o** High **o** Very high. **O** NA for my role  **Introduction to Questions 6 & 7: Design**  Protocol design may affect accessibility.  **Option for click here: Questions 6 & 7: Design**  Design factors affecting protocol accessibility include: visit duration; accommodating physical settings; virtual vs. in-person depending upon disability, tech capability, etc.; adaptive scales/exam tables; (e.g. plain language study materials; text-to-speech; read items aloud.  **Questions 6 & 7: Design**   1. Think about research protocols that you’ve been/are/will be involved with. Rate your **knowledge** of possible ways to make them more accessible to people with functional limitations   **o** Very low **o** Low. **o** Moderate **o** High **o** Very high. **O** NA for my role   1. Think about your experiences developing studies. Now, rate your **ability** to implement any such accessibility modifications:   **o** Very low **o** Low. **o** Moderate **o** High **o** Very high. **O** NA for my role  **Introduction to Questions: 8 & 9 Risk**  Most human subjects research involves some level of risk.  **Option for click here: Questions 8 & 9: Risk**  Examples include: invasive procedures (e.g., blood draws), treatment side-effects, psychological discomfort and disruption of routines. Benefits are improved individual, community or population health and scientific advances.  **Questions 8 & 9: Risk**   1. Rate your **knowledge** of any additional risks for PWI/DD participating in research studies you do, compared to their non-IDD/D counterparts.   **o** Very low **o** Low. **o** Moderate **o** High **o** Very high. **O** NA for my role   1. Rate your **ability** to minimize any such potential added risks for PWIDD   **o** Very low **o** Low. **o** Moderate **o** High **o** Very high. **O** NA for my role  **Introduction to Questions: 10 & 11 Eligibility**  Eligibility criteria are often overly broad, lack clarity, and/or lack justification.  **Option for click here: Questions 10 & 11: Eligibility**  In one study, 85% of protocols allowed broad investigator discretion (e.g., “Any other reason the investigator deems exclusionary), just 18% explicitly permitted supports for PWD/DD, and just 24% had documented justification for excluding the disability.  Strategies to *reduce* reliance on such broad investigator discretion include:   - Use of sensitive assessment tools, such as the Frailty Index vs. an individual’s age alone or Investigator judgment - Conducting a needs analysis, to inform targeted additional protections - A disability coordinator   (DeCormier Plosky, Ne'eman et al. 2022)  **Questions 10 & 11: Eligibility**   1. Many research studies exclude PWIDD. Think about the eligibility restrictions in your research studies and whether they are likely to exclude PWI/DD. Rate your **knowledge** of the evidence for these restrictions that would likely exclude PWI/DD. (DeCormier Plosky, Ne'eman et al. 2022)   Very low **o** Low. **o** Moderate **o** High **o** Very high. **O** NA for my role   1. Rate your **ability** to integrate skills/ strategies/tools that better define, narrow, and justify your study’s eligibility restrictions.   **o** Very low **o** Low. **o** Moderate **o** High **o** Very high. **O** NA for my role  **REINFORCEMENT (R)** | |  |
|  |  |  |
| **Questions 12A, B, C: Reinforcement** |  |  |
| Rate how much impact the strategies below would likely **reinforce** your inclusion of PWD/DD in research (i.e., conceptualization, recruitment, design):  **12A)** If human subjects committees required IRB applications to report disability demographics for the proposed disease or condition or research area?  **o** Very low **o** Low. **o** Moderate **o** High **o** Very high. **O** NA for my role  **12B)** If journal editors required that manuscript submissions report disability demographics for study samples?  **o** Very low **o** Low. **o** Moderate **o** High **o** Very high. **O** NA for my role  **12C)** If federal funders required a “Inclusion of Persons with Disabilities” attachment to the application?  **o** Very low **o** Low. **o** Moderate **o** High **o** Very high. **O** NA for my role | |  |
| **Introduction to Questions 13A, B, C: Disability as Diversity**  In September 2023, the NIH designated people with disabilities a population with health disparities. ([click here to learn more](https://www.nih.gov/news-events/news-releases/nih-designates-people-disabilities-population-health-disparities))  **Questions 13A, 13B & 1C**  What actual impact, if any, will this designation have on:  13A) Learning how your areas of research (e.g., diabetes, cancer screening) impact PWD/DD (ex. differential prevalence, complications, and/or treatment for PWI/DD vs. without  **o** Very low **o** Low. **o** Moderate **o** High **o** Very high. **O** NA for my role  13B) Considering PWD/DD as suitable research participants?  **o** Very low **o** Low. **o** Moderate **o** High **o** Very high. **O** NA for my role  13C) Recruiting PWD/DD for your research?  **o** Very low **o** Low. **o** Moderate **o** High **o** Very high. **O** NA for my role | |  |
